# METTL16‐mediated N6‐methyladenosine modification of *Soga1* enables proper chromosome segregation and chromosomal stability in colorectal cancer

**DOI:** 10.1111/cpr.13590

**Published:** 2023-12-12

**Authors:** Jimin Li, Fang Yang, Zeyu Wang, Siqing Zheng, Shuang Zhang, Chen Wang, Bing He, Jia‐Bei Wang, Hao Wang

**Affiliations:** ^1^ Department of Laboratory Medicine The Affiliated Anhui Provincial Hospital of Anhui Medical University Hefei China; ^2^ Department of Clinical Laboratory The First Affiliated Hospital of Wannan Medical College (Yijishan Hospital of Wannan Medical College) Wuhu China; ^3^ Graduate School, Bengbu Medical College Bengbu China; ^4^ School of Pharmacy Anhui Medical University Hefei China; ^5^ Department of Hepatobiliary Surgery, Anhui Province Key Laboratory of Hepatopancreatobiliary Surgery, The First Affiliated Hospital of USTC, Division of Life Sciences and Medicine University of Science and Technology of China Heifei China

## Abstract

N6‐methyladenosine (m6A) is the most prevalent internal modification in mammalian messenger RNAs and is associated with numerous biological processes. However, its role in chromosomal instability remains to be established. Here, we report that an RNA m6A methyltransferase, METTL16, plays an indispensable role in the progression of chromosome segregation and is required to preserve chromosome stability in colorectal cancer (CRC) cells. Depletion or inhibition of the methyltransferase activity of METTL16 results in abnormal kinetochore‐microtubule attachment during mitosis, leading to delayed mitosis, lagging chromosomes, chromosome mis‐segregation and chromosomal instability. Mechanistically, METTL16 exerts its oncogenic effects by enhancing the expression of suppressor of glucose by autophagy 1 (*Soga1*) in an m6A‐dependent manner. CDK1 phosphorylates Soga1, thereby triggering its direct interaction with the polo box domain of PLK1. This interaction facilitates PLK1 activation and promotes mitotic progression. Therefore, targeting the METTL16‐Soga1 pathway may provide a potential treatment strategy against CRC because of its essential role in maintaining chromosomal stability.

## Graphical abstract

METTL16 catalyses the addition of m6A to *Soga1* messenger RNA (mRNA) transcripts, leading to stabilization and increased levels of *Soga1* mRNA through IGF2BP1‐mediated mechanisms. CDK1‐mediated phosphorylation of Soga1 generates a PLK1 docking site that promotes PLK1 activation. This further promotes the activation of Aurora B kinase, which mediates erroneous kinetochore‐microtubule attachments. These results indicate that METTL16 promotes colorectal cancer proliferation by activating the METTL16/m6A/Soga1 axis.

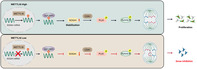

## 1 INTRODUCTION

Colorectal cancer (CRC) ranks as the fourth most common malignant tumour in humans and is the third most common cause of cancer‐related mortality worldwide.^1^ In recent years, significant advances in surgical treatment and adjuvant therapy have improved the prognosis of CRC patients; however, high mortality rates persist because of inadequate early diagnostic methods and a lack of effective interventions. Therefore, a better understanding of the mechanisms underlying the development and progression of CRC is important to enable identification of novel diagnostic biomarkers and therapeutic targets.

Genome instability is a prominent pathogenic feature and one of the earliest molecular events of cancer.^2^, ^3^ Chromosome instability (CIN) is the most common form of genome instability and is characterized by high levels of aberrations in chromosome structure and number.^4^, ^5^ By continuously generating novel aneuploid genomes, CIN contributes to the heterogeneity of cancer cells, which enables clonal selectivity. A primary cause of CIN is asymmetric segregation of metaphase chromosomes caused by abnormal spindle formation, resulting in anaphase bridging during mitosis, a hallmark feature of CIN.^2^, ^6^, ^7^ CIN is implicated in poorer survival, metastasis and therapeutic resistance in CRC,^4^, ^8^ but the underlying molecular mechanisms remain poorly understood.

RNA N6‐methyladenosine (m6A) is the most prevalent mRNA modification in mammals and plays a pivotal function in the post‐transcriptional regulation of messenger RNA (mRNA) splicing.^9^, ^10^ m6A modification is a dynamic and reversible event catalysed by m6A methyltransferases (METTL3, METTL14, WTAP and METTL16) and removed by the demethylases, FTO and ALKBH5.^11^, ^12^ In addition, RNA binding proteins (YTHDF1/2/3, IGF2BP1/2/3, HNRNPC) recognize and bind to m6A motifs to control RNA metabolism.^13^ m6A modification is involved in various physiological and pathological processes, such as stem cell differentiation, DNA damage, circadian rhythms, embryonic development, translation and degradation.^14^ Recently, mounting evidence has implicated m6A modulators in the initiation and malignant development of diverse tumour types.^10^, ^15^, ^16^ Nevertheless, the precise role and underlying mechanisms of m6A modification in CRC, particularly in relation to CIN, remain poorly understood.

In this study, we identified *METTL16* to be an essential gene for the survival of CRC cells. Specifically, METTL16 is indispensable for the proper progression of mitosis and maintains chromosome stability by fine‐tuning the expression of its crucial target, Soga1, in an m6a‐dependent post‐transcriptional manner. Our findings indicate that METTL16 has potential as a novel predictive indicator and a new therapeutic target in CRC.

## 2 MATERIALS AND METHODS

### 2.1 Cell culture and treatments

The HCT116, SW620, HEK293T and HeLa were obtained from the American Type Culture Collection (ATCC). HCT116 was cultured in McCoys, 5A (Hyclone), and HEK293T, SW620 and HeLa cells were grown in Dulbecco's modified eagle medium (Gibco). All media were supplemented with 10% fetal bovine serum. The cells were synchronized by double‐thymidine block to obtain an enriched population of M phase cells or in prometaphase with 0.33 μM nocodazole (MedChemExpress). Mitotic cells were further enriched by ‘shake‐off’.

### 2.2 Plasmid construction and transfection

Full‐length METTL16, METTL16‐PP185/186AA and Soga1 were cloned into the pcDNA3.1‐FLAG plasmids or PLVX‐CMV‐puro lentiviral plasmids. All constructs used in this study were verified by DNA sequencing. For transfection, siRNA or plasmids were transfected into cells using Lipofectamine 2000 reagent (Thermo Fisher Scientific) according to the manufacturer's instructions, and the sequences of indicated siRNA/shRNA are shown in Table S1.

### 2.3 Lentivirus packaging and infection

For virus packaging and lentiviral infection, lentiviruses were produced in HEK293T cells by co‐transfection with pMD2G and pSPAX2. Forty‐eight hours after transfection, infectious lentiviruses were collected and were used for the transduction of indicated cells in the presence of polybrene (Sigma).

### 2.4 Co‐immunoprecipitation and Western blotting

Cells were lysed using lysis buffer (20 mM Tris–HCl, pH 8.0, 100 mM NaCl, 0.5% NP‐40 and cocktail protease inhibitors). The cell lysate was incubated with indicated antibodies at 4°C overnight, followed by the addition of protein G‐coated agarose beads (Invitrogen). After incubation at 4°C for 4 h, beads were washed three times with the lysis buffer. The precipitated components were eluted by boiling the beads in Sodium dodecyl sulfate loading buffer. Western blotting was performed as previously described. Primary antibodies were listed as: METTL16 (Sigama, HPA020352), METTL16 (abconal, A15894), Soga1 (Santa, sc‐514,885), GAPDH (abconal, AC001), anti‐p‐Ser/Thr (BD Biosciences, 612,549), PLK1 (abcam, ab17057), PLK1‐pT210 (abcam, ab155095), Flag (CST, 14793), Flag (Sigma, F3165), HA (abconal, AE008), HA (abconal, AE036).

### 2.5 Quantitative real‐time polymerase chain reaction and RNA stability assay

Total RNA of cells was extracted by TRIzol Regent (Invitrogen) according to the previous protocols followed by cDNA synthesis with PrimeScript™ RT Master Mix (RR036A, TAKARA). mRNA expression levels were measured by TB Green® Premix Ex Taq™ II Kit (RR820A, TAKAR) on Applied Biosystems StepOnePlus. Relative RNA amount of each group was calculated using the 2^−ΔΔCt^ method with normalization by GAPDH.

For RNA stability assay, cells were seeded in six‐well plates overnight, RNA decay rate was measured using actinomycin D (HY‐17559, MedChemExpress) at 5 μg/mL and cells were harvested after incubation at indicated time. The primers used in this study are presented in Table S2.

### 2.6 Methylated RNA immunoprecipiation sequencing (MeRIP‐sequencing)

Total RNA was extracted from SW620 cells transfected with shMETTL16‐2 or shNC using TRIZOL reagent, followed by poly (A) + RNA purification and fragmentation using the NEBNext poly (A) mRNA Magnetic Isolation Kit (New England Biolabs, UK). MeRIP‐sequencing and data analysis were supported by Genesky Biotechnologies Inc (Shanghai, China). Qualified samples underwent Library Pooling and Sequencing using Illumina HiSeq 2500 machines. Following quality filter, the raw sequence data were mapped to human genome GRCh37/hg19 utilizing the HISAT2 software (v2.0.5) and the results were subjected to analysed bioinformatically and statistically.

### 2.7 RNA immunoprecipitation

Cell lysates were rotary incubated with 1 μg specific antibodies against IgG, or IGF2BP1 at 4°C overnight to precipitate m6A‐conjugated RNAs in Reaction Buffer, then 20 μL washed magnetic beads was added to each reaction and incubated at 4°C for 2 h. After washed three times with Phosphate buffered saline(PBS), the target RNAs in the immunoprecipitation complex were purified for further analysis by quantitative real‐time polymerase chain reaction (qRT‐PCR). The relative enrichment of RNA was normalized to the input.

### 2.8 RNA pull‐down assay

An amount of 1 × 10^7^ cells were lysed, and the lysates were rotary incubated with 3 μg biotin‐labelled probe mixed with cocktail and ribonuclease inhibitor at 4°C overnight. Then, 20 μL of pre‐cleared streptavidin magnetic beads (88816, ThermoFisher Scientific) was added to the cell lysates to precipitate the RNA–protein complex. After elution from the beads with lysis buffer for three times, the co‐precipitated proteins were boiled with loading buffer for 10 min for further analysis by western blotting. The biotin probe sequence was listed as follows: AGCAGGAAGTTGTGCTTGAATTGCT, negative control: AGCAATTCAAGCACAACTTCCTGCT.

### 2.9 Immunohistochemistry

The CRC and adjacent normal tissue were subjected to immunohistochemistry (IHC) staining according to previous protocol. Briefly, following deparaffinization, rehydration and antigen retrieval, sections were conjugated with primary antibodies at 4°C overnight. After incubation with secondary and development of diaminobenzidine, the mean density of target proteins were analysed using ImageJ.

### 2.10 Immunofluorescence and live‐cell imaging

Cells were fixed with 1× PBS buffer containing 3.7% paraformaldehyde for 10 min and then permeabilized with 0.5% Triton X‐100 for 10 min at room temperature. After blocked with 5% bovine serum albumin  (in 1× PBS) for 1 h, the cells incubation with primary antibodies overnight at 4°C. After washing with PBS, the second antibody was added, and the slides were kept at room temperature for 2 h. Cells were washed with 1× PBS three times and then incubated with 4,6‐diamidino‐2‐phenylindole (DAPI) at room temperature for 3 min. After washing with 1× PBS three times, the glass placed with cells was put upside‐down on a glass slide and sealed with ProLong TM Gold antifade reagents (Thermo Fisher Scientific) prior to microscopy observation after drying. Images were captured with a fluorescence microscope (Leika, SP8). Primary antibodies were listed as: human anti‐centromere antisera (ACA) (Antibodies Inc., 15–234‐0001), phospho‐Aurora B (Rockland, 600–401‐677), Phospho‐Histone H3S10 (CST, 9706), Phospho‐CENP‐A‐S7 (CST, 2187), INCENP (Santa, sc‐376,514), survivin (Santa, sc‐17,779), Tubulin (Proteintech, 66,031‐1‐Ig). Time‐lapse live cell imaging was carried out with the HeLa cells expressing H2B‐GFP. Cells were plated in glass‐bottom dishes and were observed on humidified environment (37°C and 5% CO_2_). Images were recorded every 3 min.

### 2.11 Chromosome spread and karyotype analysis

HCT116 cells were treated with 100 ng/mL colcemid for 3–4 h. Cells were collected and hypotonically swollen in pre‐warmed 75 mM KCl for 15 min. Cells were fixed in freshly made fixative solution (methanol: acetic acid 3:1) with 3–5 times changes of the fixative. Cells were dropped onto ice‐cold glass slides and air dried and were stained with 5% Giemsa for 5 min, gently rinsed with running water, air dried and mounted.

### 2.12 Colony formation assay and CCK‐8 assay

For colony formation assay, cells (1000) were seeded in triplicate (6‐well plates) per condition and allowed to adhere for a minimum of 16 h. After 10–14 days, plates were fixed and stained with Crystal Violet and counted. In the cell proliferation assay, 5 × 10^3^ cells/well were seeded into 96‐well plates. After the cells adhered, 10 μL of CCK‐8 reagent (Dojindo) was added to each well on 24, 48 and 72 h, the absorbance was measured at 450 nm.

### 2.13 GST pull‐down assay

Glutathione S‐transferase (GST) or GST‐fusion proteins were expressed in *Escherichia coli* induced with Isopropyl‐beta‐D‐thiogalactopyranoside. The lysate was clarified by centrifugation and incubated with Glutathione Sepharose 4B (GE Healthcare). HCT116 cells were lysed using lysis buffer (20 mM Tris–HCl, pH 8.0, 100 mM NaCl, 0.5% NP‐40 and cocktail protease inhibitors), then incubated with GST fusion proteins immobilized to glutathione Sepharose 4B beads for 8 h. The beads were washed 3–5 times with the same lysis buffer and subjected to analysis by immunoblotting or Coomassie Brilliant Blue (CBB) staining.

### 2.14 Luciferase reporter assay

Cells were transfected with siRNAs using Lipofectamine 2000 (Invitrogen). After overnight incubation, cells were further transfected with indicated reporter vectors using GeneTwin™. Then after 24 h of incubation, the luciferase activity was determined with the Dual‐Luciferase Reporter Assay system (Vazyme) according to the supplier's instructions. The relative luciferase activity was calculated by dividing Fluc by Rluc and normalized to individual control for each assay.

### 2.15 Statistical analysis

Data are presented as the mean ± SE of at least three independent experiments. The results were tested for significance using the unpaired Student's *t*‐test (**p < 0.01 and *p < 0.05 were considered significant).

## 3 RESULTS

### 3.1 METTL16 promotes CRC cell survival and proliferation

**FIGURE 1:**
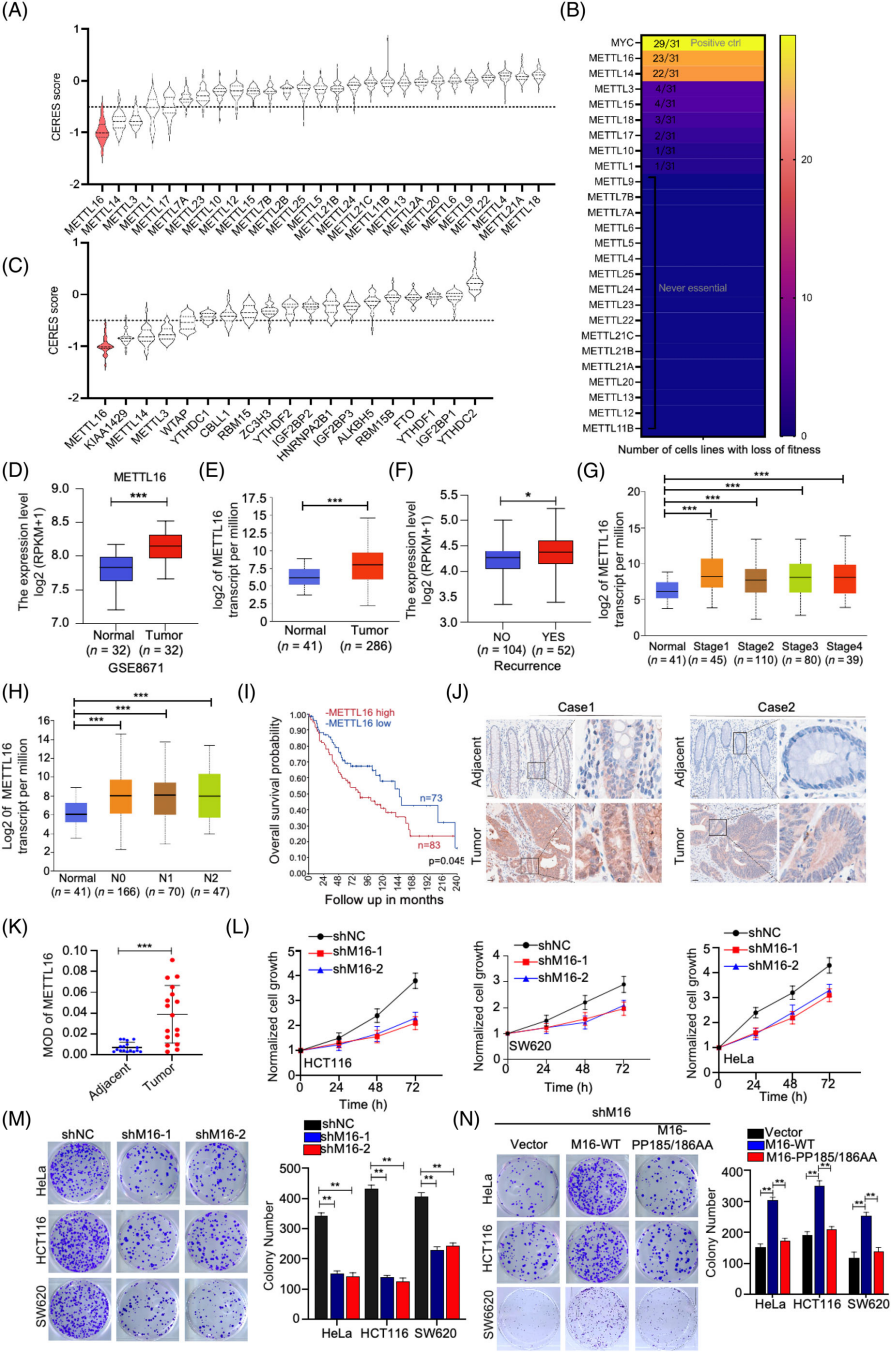
METTL16 is required for CRC cell survival and proliferation. (A, C) The CERES scores of METTL family members and m6A regulators were obtained from CRISPR‐associated protein 9 knockout screening datasets across 56 human CRC cell lines, respectively. The raw data were obtained from DepMap (https://depmap.org/portal/). CERES scores 0 and −1 represent the median effect of non‐essential genes and common core essential genes, respectively. A lower CERES score indicates a higher dependency of the specific gene on cancer progression. (B) Another set of CRISPR‐associated protein 9 knockout screening datasets were analysed across 31 human colorectal cancer cell lines. The raw data were obtained from https://score.depmap.sanger.ac.uk/. MYC was identified as the positive control, being acknowledged as a promising therapeutic target for cancer. For each gene, the number of essential functions and total CRC cell lines were displayed. For instance, METTL16 (31/23) indicates that knockout of METTL16 displays an essential function in 23 out of 31 cell lines. (D) METTL16 expression in the GSE8671 CRC database. (E) The mRNA level of METTL16 in normal tissues and CRC tissues downloaded from The Cancer Genome Atlas (TCGA) database (http://ualcan.path.uab.edu/). (F–H) Association of METTL16 mRNA expression with recurrence (F), tumour stages (G) and lymph node metastasis (H) in CRC patients in TCGA database (http://ualcan.path.uab.edu/). (I) Kaplan–Meier survival curves were generated to depict the overall survival based on METTL16 expression in GSE106584. (J–K) Representative images of METTL16 IHC staining in CRC tissues and adjacent tissues. The brown colour indicates positive staining for METTL16. MOD of METTL16 staining in CRC tissues and adjacent tissues. Scale bar: 50 μm. (L–M) Effect of METTL16 knock down on the proliferation in HCT116, SW620 and Hela cells (L, detected by CCK‐8 assay; M, detected by colony formation). (N) Rescued effects of wild‐type METTL16 and its enzymatically inactive mutants on cell proliferation of HCT116, HeLa and SW620 cells following METTL16 deletion. Error bars in this figure represent SD. *p ≤ 0.05, **p ≤ 0.01, ***p ≤ 0.001, two‐tailed *t*‐test. CRC, colorectal cancer; CRISPR, clustered regularly interspaced short palindromic repeat; IHC, immunohistochemistry; m6A, N6‐methyladenosine.

To explore novel genes essential for CRC in an unbiased manner, we analysed two large‐scale genome‐wide clustered regularly interspaced short palindromic repeat (CRISPR)‐associated protein 9 knockout screening datasets.^17^, ^18^ Among all METTL members, *METTL16* was the most critical for CRC cell survival (Figure 1A,B). Furthermore, among the major m6A regulators, *METTL16* was critical in CRC cell survival (Figures 1C and S1A). Analysis of Gene Expression Omnibus and The Cancer Genome Atlas (TCGA) databases showed that *METTL16* mRNA levels were aberrantly overexpressed in CRC samples compared with normal samples (Figure 1D,E). Furthermore, TCGA analysis revealed a strong correlation between *METTL16* levels and clinicopathological variables in CRC. Specifically, increased *METTL16* expression was associated with recurrence, advanced clinical stage and lymph node metastases (Figure 1F–H). Elevated expression of *METTL16* is significantly related to unfavourable prognosis in CRC patients (Figure 1I). In addition, immunohistochemical staining of CRC and adjacent tissues further confirmed the elevated level of METTL16 in CRC tissues (Figure 1J,K).

To explore the contribution of METTL16 to colorectal carcinogenesis, we utilized short‐hairpin RNA (shRNA)‐mediated knockdown to silence *METTL16* expression in HCT116 (CRC cell line), SW620 (CRC cell line) and HeLa cells (cervical cancer cell line) (Figure S1B). *METTL16* knockdown markedly impaired cell growth and colony formation in all three cell lines (Figure 1L,M). Furthermore, re‐expression of wild‐type *METTL16* restored cell growth in *METTL16*‐knockdown cells while enzyme‐inactivated METTL16 (PP185/186AA),^19^ did not have this effect (Figure 1N). Collectively, our findings indicate that the upregulation of *METTL16* expression promotes progression of CRC.

### 3.2 METTL16 contributes to timely progression of mitosis

**FIGURE 2:**
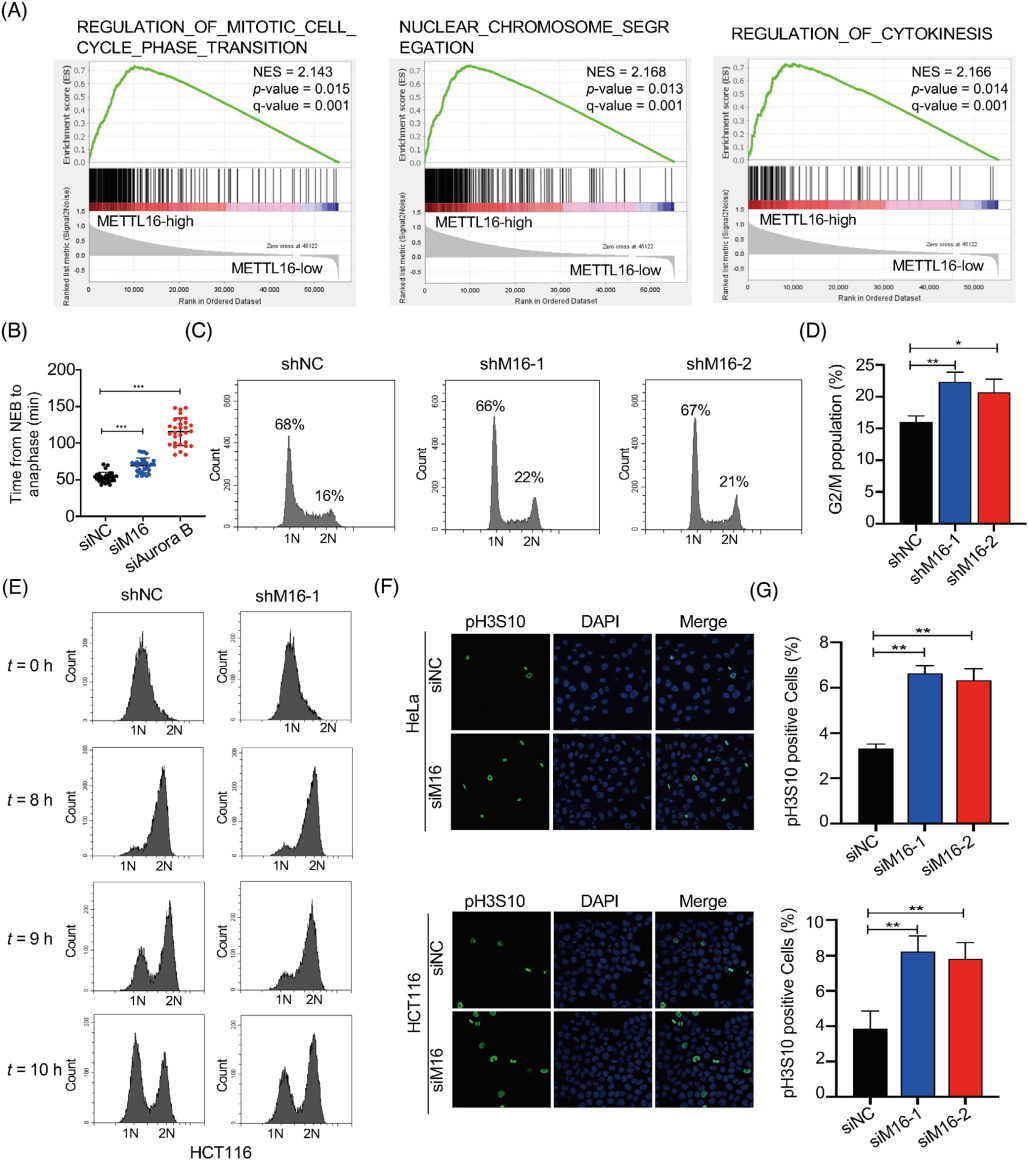
METTL16 is required for timely progression of mitosis. (A) GSEA on METTL16 CRC cancer gene signatures derived from GDC‐TCGA‐COAD plus GDC‐TCGA‐READ RNA‐seq data sets. Shown are enrichments of cell cycle, chromosome segregation and mitotic cell cycle phase transition gene sets. (B) Plot showing the average time of mitotic duration from NEB to anaphase for indicated cells. (C, D) Effect of METTL16 knock down on the cell cycle were analysed by flow cytometry. The percentage of cells with 2N and 4N is shown. (E) HCT116 cells expressing METTL16 or control shRNA were synchronized by double‐thymidine blocking treatment. Subsequently, cells released for the indicated time were analysed by FACS. (F, G) Immunofluorescence visualization of mitotic cells. NC and METTL16 deletion HeLa and HCT116 cells were stained for pH3S10 (green) and DAPI (blue). Error bars in this figure represent SD. *p ≤ 0.05, **p ≤ 0.01, two‐tailed *t*‐test. CRC, colorectal cancer; NEB, nuclear envelope breakdown; NES, normalized enrichment score; *p*‐value, nominal *p*‐value; *q*‐value, false discovery rate *q*‐value.

To uncover the mechanism of METTL16 action in CRC, we performed gene set enrichment analysis in a cohort of CRC patients (GDC‐TCGA_COAD+READ) to predict the significant signalling pathways connected to METTL16. Notably, our findings revealed a positive relationship between the expression of *METTL16* and cell cycle regulation, chromosome segregation and the mitotic cell cycle phase transition gene sets (Figure 2A). These findings led us to investigate the function of METTL16 in mitosis. To this end, we knocked down *METTL16* using siRNA in HeLa cells expressing GFP‐tagged histone H2B (widely used to visualize the dynamics of chromosomal architecture in living cells) and monitored the mitotic process using a living cell microscopy system. As shown in Figures 2B and S1B, control cells completed mitosis within ~50 min, whereas *METTL16* knockdown resulted in a prolonged mitotic phase from nuclear envelope breakdown (NEB) to anaphase, which typically lasted ~70 min. As a positive control, the Aurora B knockdown group significantly extended the duration of the mitotic phase to ~120 min.

Consistently, knockdown METTL16 led to an increase in the proportion of cells in G2/M phase as revealed by Fluorescence‐activated cell sorting (FACS) analysis (Figure 2C,D). FACS analysis also revealed mitotic delays during synchronized cell mitosis after release of the thymidine double block (Figure 2E). These findings indicate that knockdown METTL16 prolongs M phase duration. Consistently, we observed an accumulation of pH3S10‐positive cells following METTL16 knockdown (Figure 2F,G). Collectively, these findings indicate that *METTL16* knockdown results in cell accumulation at the G2/M phase.

### 3.3 METTL16 promotes accurate chromosome segregation by regulating Aurora B activation

**FIGURE 3:**
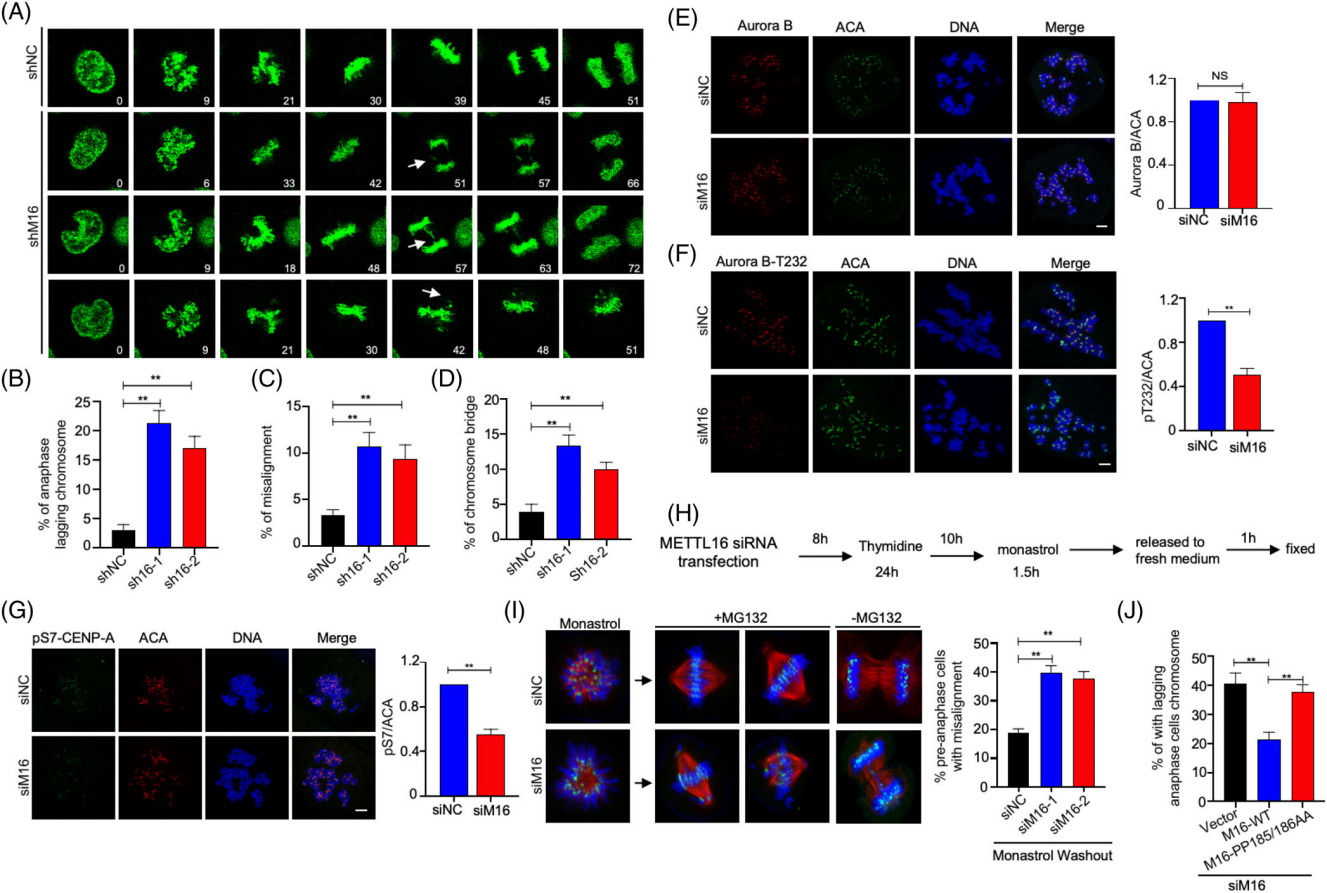
METTL16 promotes Aurora B kinase activity and attachment error correction. (A–D) Representative mitotic phenotypes in HeLa cells expressing GFP‐tagged histone H2B transfected shMETTL16 and control shRNA. (arrowheads indicate lagging chromosomes, chromosome bridges and chromosome misalignments). (E, F) Fluorescence intensity and quantification of Aurora B‐pT232 and Aurora B at centromeres in prometaphase HeLa cells transfecting the control or METTL16 siRNA. Mitotic cells were obtained after nocodazole treatment for 5 h. (G) Representative images of pS7‐CENP‐A in mitotic cells transfecting the control or METTL16 siRNA. Mitotic cells were obtained after nocodazole treatment for 5 h. (H, I) HeLa cells transfecting control siRNA or METTL16 siRNA were synchronized to mitosis by thymidine release. The mitotic cells were incubated with monastrol (50 μM) for 1.5 h to generate erroneous attachments, and cells were then released into fresh media with or without MG132 for another 1 h before fixation. Errors in chromosome segregation were quantified from anaphase cells. (J) Quantification of indicated phenotypes of METTL16‐depleted cells rescued by METTL16‐WT or METTL16‐PP185/186AA. Error bars in this figure represent SD. *p ≤ 0.05, **p ≤ 0.01, two‐tailed *t*‐test.

To further investigate the function of METTL16 in the mitotic process, we knocked down endogenous METTL16 in GFP‐H2B‐HeLa cells and monitored the subsequent changes in mitosis by time‐lapse microscopy. Whereas control cells proceeded normally through mitosis, METTL16‐deficient cells displayed considerably more chromosome segregation aberrations, such as lagging chromosomes, misalignment and chromosome bridges (Figure 3A–D).

Aurora B kinase plays a critical role in the correction of erroneous kinetochore‐microtubule attachments, which is the major contributor to lagging chromosomes^20^, ^21^; therefore, we questioned whether the function of METTL16 in promoting accurate chromosome segregation was mediated via Aurora B signalling. To achieve this, we knockdown Aurora B through siRNA treatment. While Aurora B knockdown resulted in a more pronounced phenotype compared to METTL16 knockdown, the combined knockdown of Aurora B and METTL16 did not exhibit an additive effect (Figure S2A,B). This suggests the presence of a functional relationship between these two proteins. Next, we explore whether METTL16 impacts the localization or the activity of Aurora B at centromeres. Knockdown of METTL16 barely affected the centromeric location of Aurora B in prometaphase cells following nocodazole treatment (Figure 3E). However, the phosphorylation level of Aurora B Thr232, which is an autophosphorylation site critical for full Aurora B activity,^22^ was markedly decreased in METTL16‐deficient cells (Figure 3F).

Aurora B is a constituent of the chromosomal passenger complex (CPC), which consists of inner centromere protein (INCENP), Survivin and Borealin.^21^, ^23^, ^24^, ^25^ The location and activation of Aurora B depend on CPC assembly and multiple phosphorylation events involving CPC components.^26^, ^27^, ^28^, ^29^ Immunostaining showed that METTL16 depletion did not affect the centromeric location of INCENP or Survivin (Figure S2C–E). These findings indicate that METTL16 deficiency primarily affects Aurora B activation rather than its location at centromeres. Consequently, knockdown of METTL16 led to a reduction in Aurora B‐mediated phosphorylation of CENP‐A at Ser7 (Figure 3G). To test whether the reduction in Aurora B activation at centromeres in METTL16‐deficient cells affects its capacity in correcting erroneous microtubule‐kinetochore attachments, we performed a monastrol washout assay.^30^, ^31^ In this assay, cells are arrested in monastrol to form erroneous attachments. After being released from monastrol, bipolar spindles are formed and the aberrant attachments are corrected in an Aurora B kinase‐driven manner. Eventually, chromosomes align at the metaphase plate and segregate in anaphase (Figure 3H). The proportion of cells with completely aligned or misaligned chromosomes was determined as an indicator of error correction. One hour after release into media containing MG132 (proteasome inhibitors induce mitotic cells to arrest at metaphase), ~80% of control cells achieved a fully aligned metaphase plate with no bi‐orientation defects, compared with ~55% for METTL16 knockdown cells (Figure 3I). These results indicate that METTL16 is involved in the correction of kinetochore‐microtube attachment errors. Consistently, we observed increased numbers of lagging chromosomes in the METTL16 knockdown cells as the cells entered anaphase following monastrol release (Figure S2F). Importantly, the defects observed in METTL16 knockdown cells were effectively rescued by re‐expression of wild‐type METTL16, but not of the enzymatically inactive mutant (PP185/186AA) (Figure 3J). We therefore concluded that METTL16 methyltransferase activity promotes accurate chromosome segregation, most likely by regulating Aurora B kinase activation.

### 3.4 Soga1 is an m6A‐dependent target of METTL16

**FIGURE 4:**
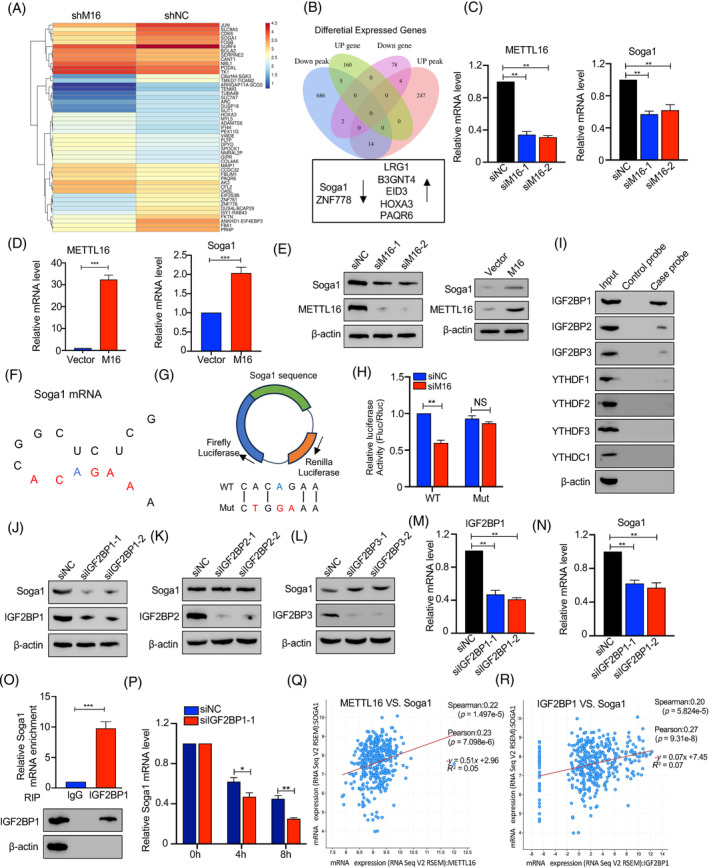
Soga1 is identified as the direct target of METTL16. (A) Heatmap showing the expression profile of differentially methylated genes after METTL16 knockdown. (B) Seven candidate target genes of METTL16 came from the intersection of RNA‐sequencing and MeRIP‐sequencing. (C, D) Soga1 mRNA expression in HCT116 cells with METTL16 knockdown or overexpression were detected by qRT‐PCR. (E) The Soga1 protein expression in HCT116 cells with METTL16 knockdown and with METTL16 overexpression were detected by western blotting. (F) Predicted secondary structures surrounding m6 peaks in Soga1 mRNA. The 5′‐ACAGAR‐3′ boxes were shown in red and the m6A modification sites were shown in blue. (G, H) pmirGLO reporter vector encoding firefly and renilla luciferase were used. Indicated wild‐type (WT) and mutant (Mut) sequences were inserted at the 3′ end of the firefly luciferase. (H) Luciferase reporter activity of indicated reporters under METTL16 knockdown in 293T cells. Renilla luciferase activity was used as an internal control. (I) Immunoblotting of IGF2BP1/2/3, YTHDC1, YTHDF1/2/3 after RNA pull down assay with cell lysate (input), biotinylated‐Soga1 (case probe), and beads only (ctrl probe). (J–N) Soga1 protein or mRNA expression in HCT116 cells transfected with siRNAs of IGF2BP1/2/3 were examined by western blotting and qPCR, respectively. (O) RIP‐qPCR displayed the relative enrichment of Soga1 mRNA in each group precipitated with lgG or IGF2BP1 antibody with the normalization to input. IP efficiency of IGF2BP1 was validated using western blotting. β‐actin was used as protein control. (P) The stability changes of Soga1 mRNAs in HCT116 cells upon IGF2BP1 knockdown. (Q, R) Correlation between the mRNA expression of METTL16, Soga1 and IGF2BP1. Error bars in this figure represent SD. *p ≤ 0.05, **p ≤ 0.01, ***p ≤ 0.001, two‐tailed *t*‐test. m6A, N6‐methyladenosine; qPCR, quantitative polymerase chain reaction; qRT‐PCR, quantitative real‐time polymerase chain reaction; RIP, RNA immunoprecipitation.

To determine the authentic targets that mediate the action of METTL16 in chromosome segregation, we performed MeRIP‐seq and RNA‐sequencing assays. MeRIP‐seq analysis revealed differential methylation at 18,812 sites, with the majority (17,211 sites) showing hypomethylation under METTL16 deficiency, consistent with its function as an m6A methyltransferase.^19^ Furthermore, RNA sequence data indicated that METTL16 deficiency caused 164 genes to be up‐regulated and 84 genes to be down‐regulated. The top 20 altered genes are presented in Figure 4A. Four differentially expressed genes and peaks overlapped (Figure 4B). Of these seven genes, *Soga1* was chosen for further functional analysis as it has been suggested to be essential for precise chromosomal segregation.^32^

Subsequent verification revealed that METTL16 knockdown resulted in reduced *Soga1* mRNA levels, whereas METTL16 over‐expression increased *Soga1* mRNA levels in CRC cells (Figure 4C,D). As expected, METTL16 also regulated Soga1 protein levels (Figure 4E). Fancm, Brca2, U6 snRNA and MAT2A mRNA contains METTL16‐target consensus sequences 5′‐ACAGAR‐3′ that are situated in structured RNA sequences.^33^, ^34^, ^35^ Therefore, we sought to determine if transcripts of *Soga1* also exhibit similar structured motifs. As shown in Figure 4F, we identified one such motif within *Soga1* in coding sequences. To determine the effect of METTL16‐dependent m6A regulation on *Soga1* expression, we constructed a luciferase reporter gene with an integrated coding sequence containing the WT or mutated m6A sites (Figure 4G). Luciferase assays showed that METTL16 knockdown largely decreased the activity of luciferase with WT, but not mutated, *Soga1* (Figure 4H). In summary, these findings indicate that METTL16 downregulation decreases the m6A level of *Soga1* mRNA, thereby reducing *Soga1* expression.

The IGF2BP proteins, YTHDC1 and YTHDF1, are capable of sensing m6A modifications and stabilizing the corresponding mRNAs^36^, ^37^, ^38^; therefore, we asked which m6A reader mediates *Soga1* methylation. To this end, we performed an RNA pulled‐down assay to capture *Soga1*‐interacting readers. As shown in Figure 4I, *Soga1* mRNA preferentially bound to IGF2BP1/2/3. Notably, IGF2BP1 knockdown, but not IGF2BP2/3 knockdown, significantly reduced Soga1 mRNA and protein levels in CRC cells (Figure 4J–N). Moreover, the results of the RNA immunoprecipitation (RIP) assay further confirmed that IGF2BP1 directly binds to *Soga1* mRNA (Figure 4O). As expect, we observed that the binding of IGF2BP1 to *Soga1* decreased following METTL16 knockdown (Figure S3A and S3B). As expected, RNA stability assays showed that IGF2BP1 knockdown significantly decreased the stability of *Soga1* mRNA and downregulated its abundance (Figure 4P). Interesting, we found that knockdown of METTL16 alone and concurrent knockdown of METTL16 and IGF2BP1 impair *Soga1* transcript levels to similar extents, indicating that METTL16 and IGF2BP1 function in a common process in regulating *Soga1* transcript levels (Figure S3C).

The TCGA and Clinical Proteomic Tumor Analysis Consortium (CPTAC) databases showed that *IGF2BP1* expression was markedly increased in CRC samples compared with normal samples (Figures S3D,E). Furthermore, increased *IGF2BP1* expression was significantly correlated with clinical stage and lymph node metastasis (Figures S3F,G). Further analysis revealed a positive correlation between METTL16 and *IGF2BP1* mRNA levels with *Soga1* mRNA in CRC (Figure 4Q,R). Collectively, our results indicate that METTL16 deposits the m6A modifications in *Soga1* transcripts, which are recognized by IGF2BP1 to stabilize the targeted mRNA in CRC.

### 3.5 The METTL16/m6A/Soga1 axis ensure accurate mitosis

Soga1 can promote faithful chromosome segregation in human cells.^32^ Therefore, we sought to examine the function of the METTL16/m6A/Soga1 axis in promoting accurate chromosome segregation.

**FIGURE 5:**
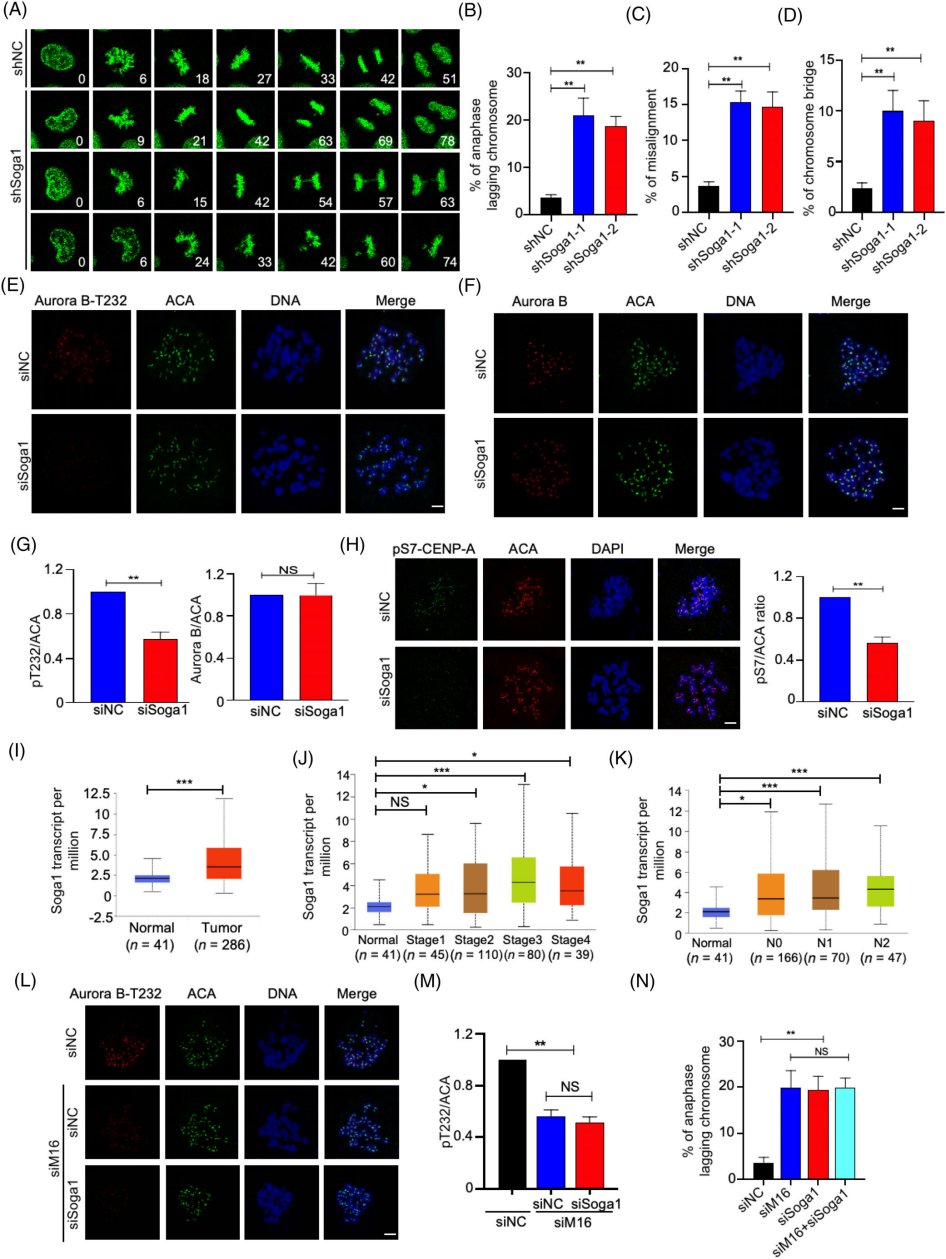
The METTL16/m6A/Soga1 axis promote chromosome segregation. (A–D) Quantification of indicated phenotypes of Soga1‐knockdown HeLa cells. (E–G) Fluorescence intensity and quantification of Aurora B‐pT232 and Aurora B at centromeres in prometaphase HeLa cells transfecting the control or Soga1 siRNA. Mitotic cells were obtained after nocodazole treatment for 5 h. (H) Representative images of pS7‐CENP‐A in mitotic cells transfecting the control or Soga1 siRNA. Mitotic cells were obtained after nocodazole treatment for 5 h. (I) The levels of Soga1 expression were analysed in CRC and normal tissues using TCGA data (http://ualcan.path.uab.edu/). (J, K) Association of Soga1 mRNA expression with tumour stages and lymph node metastasis in CRC patients in TCGA database (http://ualcan.path.uab.edu/). (L, M) Immunofluorescence assay was conducted to measure Aurora B‐pT232 of siSoga1 cells transfected with METTL16 siRNA. (N) Percentage of anaphases from indicated mitotic cells that display lagging chromosomes. Error bars in this figure represent SD. *p ≤ 0.05, **p ≤ 0.01, ***p ≤ 0.001, two‐tailed *t*‐test. CRC, colorectal cancer; m6A, N6‐methyladenosine.

*Soga1* was knocked‐down first (Figure S4A), and subsequent analysis revealed a marked elevation in mitotic errors in *Soga1*‐knockdown cells, including an approximately sevenfold increase in the frequency of anaphase lagging chromosomes, an approximately fivefold increase in chromosome misalignment, and an approximately threefold increase in the occurrence of chromosome bridges (Figure 5A–D). The defects in the Soga1 knockdown cells were largely rescued by re‐expressing an wild‐type Soga1 (Figure S4B). Notably, we found that the abundance of pT232‐Aurora B in Soga1 knockdown cells was reduced to 57% of that in control cells, whereas the level of Aurora B remained unchanged (Figures 5E–G and S4C–E). Consequently, Aurora B‐dependent centromeric CENP‐A phosphorylation at Ser7 (pS7‐CENP‐A) was also decreased in *Soga1* siRNA cells (Figure 5H). This is in line with the phenotypes observed in METTL16 knockdown cells. In addition, the expression of *Soga1* mRNA was markedly increased in CRC samples compared with normal samples. Furthermore, a correlation was found between increased *Soga1* expression and clinical stage as well as lymph node metastasis (Figure 5I–K).

Next, we aimed to investigate the essential role of Soga1 in the induction of mitotic aberration phenotypes caused by METTL16 knockdown. To achieve this goal, we knockdown Soga1 alone or simultaneously knockdown both Soga1 and METTL16 using siRNA. As shown in Figure 5L, knockdown of METTL16 and Soga1 in combination did not have an additive impact on Aurora B activation (Figure 5L,M), indicating that these two proteins function through a shared pathway to maintain accurate chromosome segregation. Consistently, knockdown of Soga1 alone or concomitant knockdown of Soga1 and METTL16 resulted in similarly severe mitotic aberration phenotypes (Figure 5N). Furthermore, overexpression of Soga1 can partly reversed METTL16 mediated‐mitotic aberration (Figure S4F). These results indicate that the METTL16/m6A/Soga1 axis is critical for promoting accurate chromosome segregation in CRC.

### 3.6 Role of Soga1 phosphorylation by CDK1 in Aurora B Activation

**FIGURE 6:**
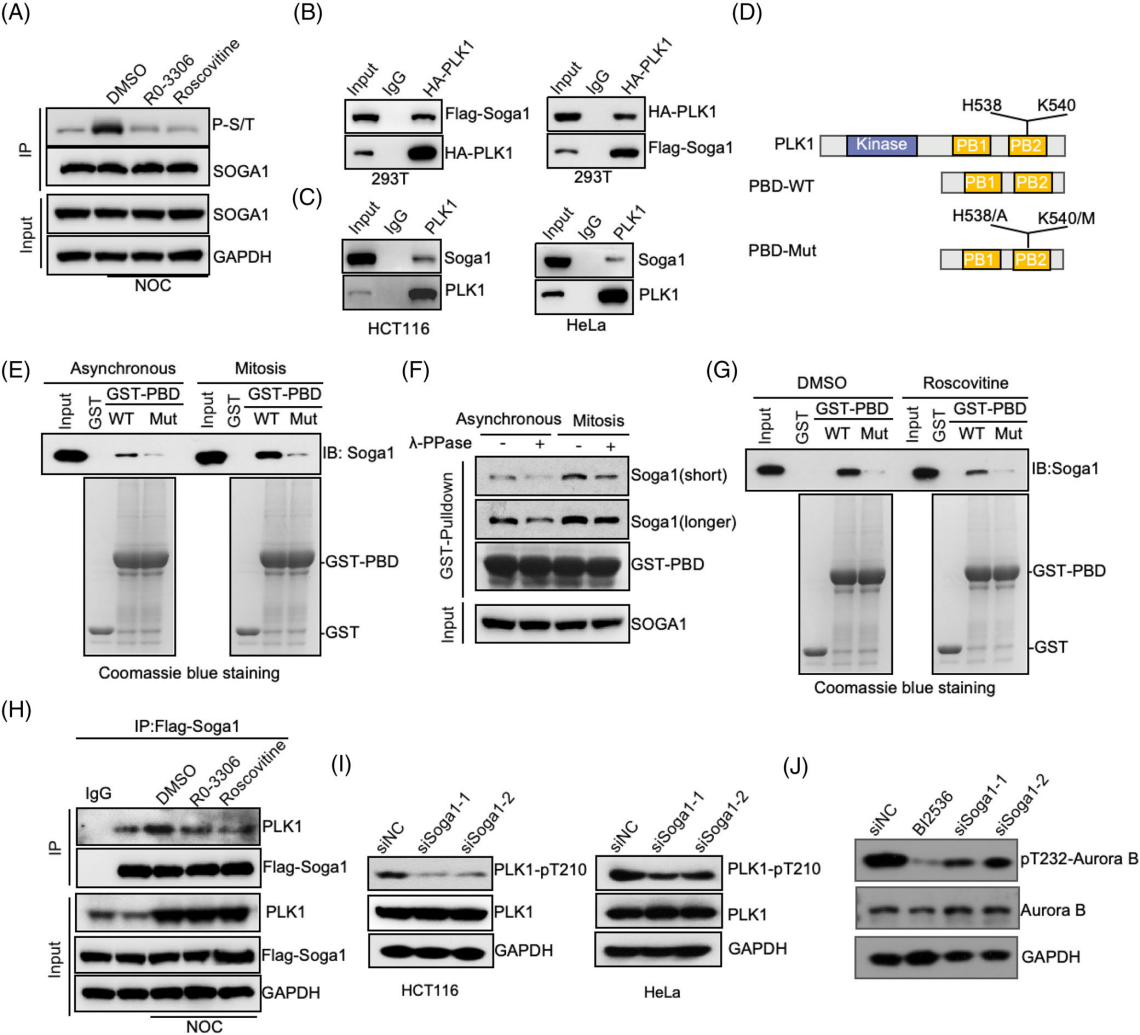
Soga1‐PLK1 axis is required for Aurora B kinase activity. (A) Immunoprecipitate for Soga1 proteins in HCT116 cells with indicated modifications or treatments. (B) HEK293T cells were transfected with a Flag‐Soga1 together with HA‐PLK1. Cell lysates were used for immunoprecipitation with Flag or HA antibodies. (C) Cell lysates from HCT116 or HeLa cells were used for immunoprecipitation with PLK1 or control IgG antibodies followed by immunoblotting. (D) Schematic description of full length PLK1 and PBD mutant. (E) Asynchronous or nocodazole‐arrested mitotic cells were subjected to pull‐downs using GST‐PBD (WT or mutant) and controls, followed by immunoblotting with Soga1 antibodies and CBB staining. (F) Lysate of asynchronous or nocodazole‐arrested mitotic cells was subjected to lambda protein phosphatase treatment and GST‐PBD pull‐downs, followed by immunoblotting with Soga1 antibodies and CBB staining. (G) Nocodazole‐arrested mitotic cells treated with roscovitine were subjected to GST‐PBD pull‐downs, followed by immunoblotting and CBB staining. (H) Unperturbed and nocodazole‐arrested cells, pre‐treated with or without the indicated drugs, were lysed and subjected to immunoprecipitation followed by western blotting with the indicated antibodies. (I) HCT116 and HeLa cells transfected with the control or Soga1 siRNA. The resulting mitotic cell lysates were subjected to immunoblotting using the indicated antibodies. (J) The HCT116 cell lysates were obtained by transfecting control or Soga1 siRNA, and treating control siRNA cells with BI2536, a PLK1 inhibitor, followed by nocodazole treatment for 16 h. The resulting mitotic cell lysates were subjected to immunoblotting using the indicated antibodies. CBB, Coomassie Brilliant Blue; Longer, long exposure; Short, short exposure.

We further investigated the underlying mechanisms involved in Soga1‐mediated regulation of Aurora B activity. CDK1 is a kinase that regulates Soga1 during mitosis.^32^ Consistent with this, more Soga1 was phosphorylated at p‐serine/threonine (p‐S/T) in nocodazole‐arrested cells than in asynchronized cells. Treatment with the CDK1 inhibitors, RO‐3306 or roscovitine, prevented this phosphorylation, indicating the specificity of CDK1‐dependent phosphorylation (Figure 6A). A phospho‐proteomics study has shown Soga1 to be phosphorylated at multiple serine and threonine residues during mitosis.^39^ Interestingly, some of the Soga1 phosphorylation sites are well matched to S[pS/pT]X, the predicted binding site for the polo box domain (PBD) of PLK1.^40^, ^41^, ^42^ To investigate the potential association between Soga1 and PLK1, we first conducted a co‐immunoprecipitation assay in HEK293T cells transfected with HA‐PLK1 and Flag‐Soga1. We observed a significant association between Soga1 and PLK1 (Figure 6B). In addition, we observed a reciprocal co‐immunoprecipitation of endogenous Soga1 and PLK1 in both HCT116 and HeLa cells (Figure 6C).

We then performed GST pull‐down assays and observed that the GST‐fused PBD exhibited significantly enhanced binding affinity for Soga1 in mitotic cell lysates compared with asynchronous cell lysates (Figure 6D,E). Given the critical role of His538 and Lys540 residues within PLK1‐PBD for phosphopeptide recognition, we generated double mutations (H538A and K540M) in PLK1‐PBD to abolish its phosphopeptide‐binding activity.^42^ Interaction studies showed that PLK1‐PBD‐H538A/K540M prevented GST‐PBD from binding to Soga1 (Figure 6D,E), indicating that the interaction between PLK1 and Soga1 relies on phosphorylation. Indeed, lambda‐phosphatase pretreatment impaired the pulldown of Soga1 by GST‐PBD in mitotic cell lysate (Figure 6F). The PBD domain of PLK1 typically binds to phosphopeptides that are driven by CDK1. Notably, the ability of Soga1 from roscovitine‐treated mitotic cells to bind GST‐PBD was significantly impaired (Figure 6G), indicating that CDK1‐dependent phosphorylation is important for the association of Soga1 with PLK1. Co‐immunoprecipitation assays were then performed to further verify this association. As shown in Figure 6H, the association between Soga1 and PLK1 was increased in nocodazole‐arrested mitotic cells, whereas this interaction was attenuated upon treatment with RO‐3306 or roscovitine. Notably, following the mutation of all 12 serine phosphorylation sites and 1 threonine site that were previously identified through mass spectrometry as sites of phosphorylation during mitosis in Soga1, changing them to alanine, we observed a reduction in its binding affinity with PLK1 in comparison to the wild‐type (Figure S5A).

The interaction between Soga1 and PLK1 led us to investigate the possible role of Soga1 in PLK1 activation. Upon knockdown of *Soga1* using two different siRNAs, we observed a reduction in pT210‐PLK1 in nocodazole‐arrested mitotic cells (Figure 6I). PLK1 kinase is important for Aurora B kinase activity^43^, ^44^, ^45^ and, consistent with this, treatment with a PLK1 inhibitor (BI2536) resulted in a significant reduction in pT232‐Aurora B (Figure 6J). Importantly, although PLK1 inhibitor caused a more severe phenotype than did Soga1 knockdown, there was no additive effect when PLK1 and Soga1 were suppressed in combination (Figure S5B), revealing that Soga1 knockdown leads to a reduction in Aurora B activity may primarily through PLK1 regulation. Furthermore, transfection of Soga1 siRNA resulted in a reduction of pT232‐Aurora B, which was rescued by transfection of Soga1‐WT but not Soga1‐13A (Figure S5C). Consistently, transfection of Soga1‐WT but not Soga1‐13A into Soga1 siRNA‐transfected cells could compromise the mitosis aberration of Soga1 knockdown (Figure S4B). Taken together, these results support the conclusion that the CDK1‐dependent phosphorylation of SOGA1 is important for regulation of Aurora B.

### 3.7 The METTL16/m6A/Soga1 axis is required for chromosomal stability

**FIGURE 7:**
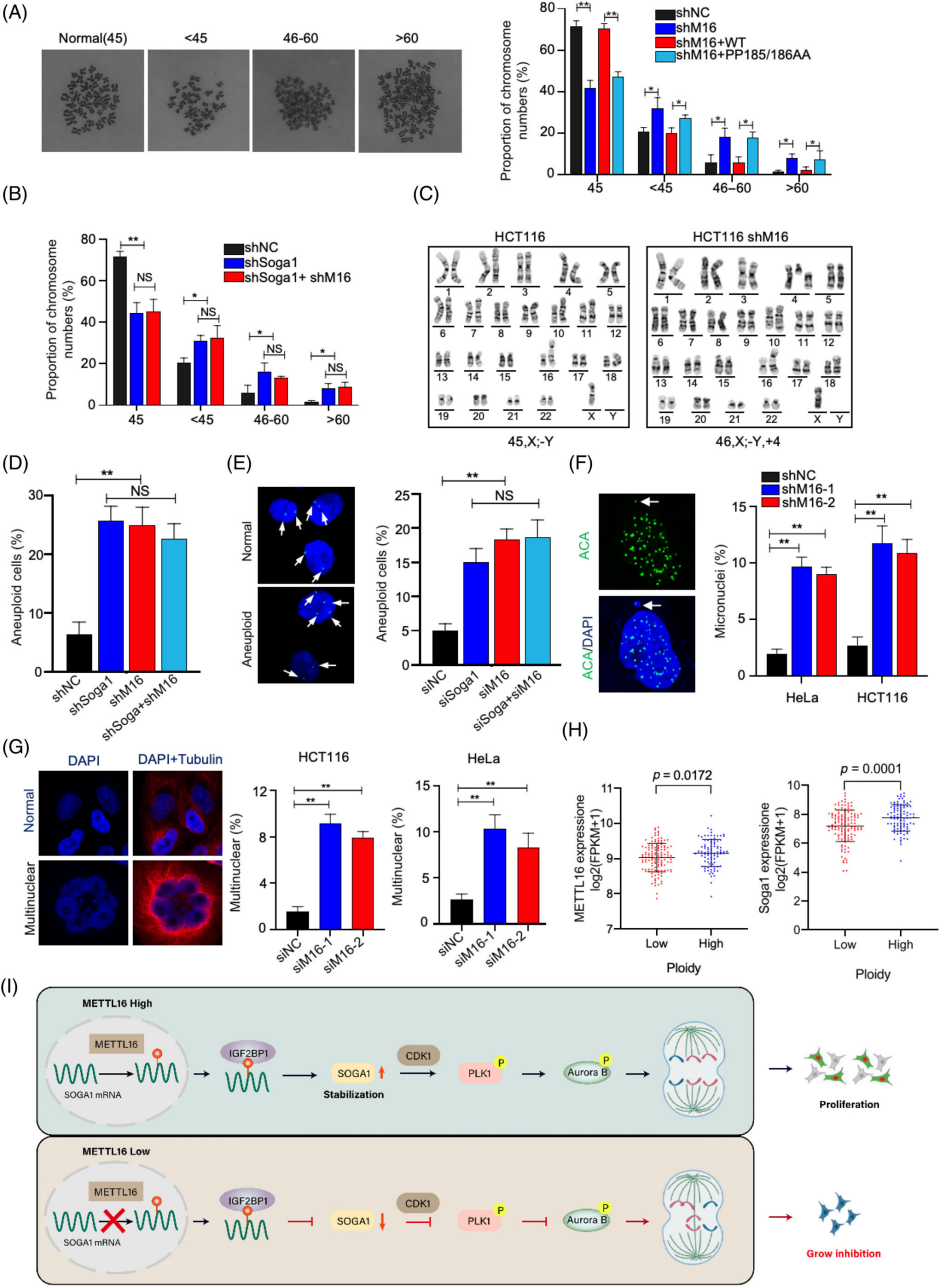
Disruption of the METTL16/m6A/Soga1 axis induces chromosomal instability. (A) A set of shRNA‐resistant rescue forms of METTL16 plasmids (WT or PP185/186AA) were stable transfected into HCT116‐shMETTL16 cells. Chromosome numbers from individual metaphase spreads were counted. (B) The downregulation with Soga1 shRNA and/or METTL16 shRNA induced aneuploidy. Chromosome numbers from individual metaphase spreads were counted. (C, D) The karyotype of HCT116 with reduced METTL16 and/or Soga1 expression was analysed. (E) Cells with reduced METTL16 and/or Soga1 expression, and analysed using Chromosome 7 alpha satellite FISH probes (green). Representative images of aneuploid cells (left) and quantification of aneuploid cells (right) are shown. (F) Percentage of micronuclei cells expressing control or METTL16 shRNA. Micronucleus stained for centromeres (ACA) and DNA. (G) HeLa cells and HCT116 cells were transfected with METTL16 siRNA or control siRNA. Immunofluorescence staining was performed with anti‐tubulin and DAPI. (H) Correlation between METTL16 expression levels and ploidy. (I) A working model showing the METTL16/m6A/Soga1 in promoting proper chromosome segregation and proliferation. *p ≤ 0.05, **p ≤ 0.01, two‐tailed *t*‐test. m6A, N6‐methyladenosine.

Chromosomal segregation defects inevitably result in aneuploidy, which can be associated with the onset and progression of tumours.^4^, ^46^ We first examined the chromosome number in chromosomally stable HCT116 cell lines. The control cells exhibited a relatively consistent karyotype (~72% normal karyotype with 45 chromosomes), while the level of aneuploidy was found to be high in METTL16‐depleted cells (~35%) (Figure 7A). Notably, this was partially rescued by re‐expression of wild‐type METTL16 but not by kinase‐impaired METTL16 (PP185/186AA) (Figure 7A).

We next tested the effect of Soga1 on METTL16‐mediated chromosomal stability. Knockdown of Soga1 in HCT116 cells caused a marked increase of aneuploidy (Figure 7B). In Soga1‐deficient CRC cells, METTL16‐deficient cells were incapable of further elevating the level of aneuploidy (Figure 7B), indicating that METTL16 promotes chromosomal stability mainly via Soga1. Detailed karyotype and Fish analyses confirmed the aneuploidy in shMETTL16 and shSoga1 cells (Figure 7C–E).

Micronuclei frequency is commonly used to evaluate chromosomal instability.^47^ Cells lacking METTL16 showed a marked elevation in micronuclei frequency relative to controls (Figure 7F). In addition, we observed an increase in giant multiple nuclear cells upon knockdown of METTL16 or Soga1 in HeLa and HCT116 cells (Figure 7G). TCGA data analysis indicates that the expression of METTL16 and Soga1 is positively correlated with ploidy (Figure 7H). Collectively, our results demonstrate that the METTL16/m6A/Soga1 axis is required for chromosome stability.

## 4 DISCUSSION

The correlation between CIN and survival has been established in CRC and in several other types of cancer.^4^, ^48^, ^49^, ^50^ An in‐depth comprehension of the molecular mechanisms underlying CIN is therefore crucial for the identification of novel treatment approaches. Here, we show that METTL16 is a key regulator of proliferation and mitotic processes in CRC. Mechanistically, METTL16 catalyses the addition of m6A to *Soga1* mRNA transcripts, leading to stabilization and increased levels of *Soga1* mRNA through IGF2BP1‐mediated mechanisms. CDK1‐mediated phosphorylation of Soga1 generates a PLK1 docking site that promotes PLK1 activation. This further promotes the activation of Aurora B kinase, which mediates erroneous kinetochore‐microtubule attachments. These results indicate that METTL16 promotes CRC progression by activating the METTL16/m6A/Soga1 axis (Figure 7I).

METTL16 was recently identified as an m6A methyltransferase, whose targets include U6 RNA, MALAT1 and MAT2A.^34^, ^35^, ^51^, ^52^ Elevated expression of METTL16 has been shown in multiple malignancies, such as hepatocellular carcinoma, lung cancer and gastric cancer, which indicates that METTL16 is broadly important in cancer.^19^, ^53^, ^54^, ^55^ Several cancer‐associated mechanisms have been proposed for METTL16. For example, METTL16 promotes gastric cancer proliferation through induction of cyclinD1 expression.^53^ In addition, METTL16 exhibits an m6A‐independent role to facilitate translation that promotes hepatoma carcinoma tumorigenesis and lung tumorigenesis.^19^, ^55^ Our study reveals a specific function of METTL16 in CRC mitosis and tumorigenicity via regulation of Aurora B kinase activity and kinetochore‐microtubule attachments. Silencing METTL16 results in prolonged mitotic time and defective chromosome segregation, characterized by lagging chromosomes, chromosome misalignment and chromosome bridges. Further investigation revealed that the methyltransferase property of METTL16 is required for proper chromosome segregation, as inactive forms of METTL16 (P185A/P186A) were unable to rescue the mitotic aberrations observed in cells lacking METTL16. It will be of great interest to identify additional substrates of METTL16 involved in mitosis and to elucidate their precise molecular functions.

Integrative MeRIP‐seq and RNA‐seq analysis identified Soga1, as a CLASP‐interacting protein required for accurate chromosome segregation during mitosis.^32^, ^56^ *Soga1* is also the most repressed gene in METTL16‐depleted cells. Our findings indicate that METTL16 directly binds to *Soga1* mRNA, inducing m6A modification and increasing mRNA stability, thereby promoting the production of Soga1 protein. In addition, RNA pulldown and RIP assays showed that IGF2BP1, but not the other readers, could bind to *Soga1* mRNA and mediate the level of Soga1 protein, indicating that IGF2BP1 is a critical reader that mediates *Soga1* m6A modification in CRC. Soga1 is a relatively uncharacterized protein; however, it is known as an autophagy suppressor and has been implicated in adiponectin‐mediated inhibition of glucose production.^57^, ^58^ Furthermore, Soga1 has a critical role in the spindle assembly checkpoint and correct chromosomal segregation.^32^ Our results confirmed these observations and further demonstrated that Soga1 plays a role in the full activation of Aurora B, which controls chromosome segregation via the generation of spindle assembly checkpoint signals and the correction of aberrant kinetochore‐microtubule attachments. Furthermore, we found that CDK1‐mediated phosphorylation primes Soga1 to associate with the PBD of PLK1, thereby promoting PLK1 activation. PLK1 can phosphorylate Aurora B, INCENP and Survivin, and this PLK1‐induced phosphorylation on the CPC component is critical for activating Aurora B. Therefore, it appears that the Soga1‐PLK1/Aurora B cascade promotes accurate chromosome segregation by facilitating Aurora B kinase activity. However, the detailed mechanism by which CDK1 phosphorylates Soga1 at precise sites remains to be elucidated.

In conclusion, our findings indicate that METTL16 has a tumour‐promoting effect in the progression of CRC. METTL16 was upregulated in CRC tissues and is associated with poor prognosis. Mechanistically, the METTL16/m6A/Soga1 signalling axis promotes CRC progression by inducing mitotic activity and malignancy. Furthermore, our data indicate that Soga1, as a mitosis‐related protein, has a role in the regulation of PLK1 and Aurora B kinase activity, independent of its glycolytic activity. These findings provide novel insights into potential diagnostic biomarkers and therapeutic targets for CRC.

## AUTHOR CONTRIBUTIONS

Hao Wang, Jia‐bei Wang and Jimin Li designed research; Jimin Li, Fang Yang, Zeyu Wang, Siqing zheng, Shuang Zhang, Chen Wang, Binbg He and Jia‐bei Wang performed or guided research; Jimin Li Fang Yang and Hao Wang analyzed data; and Jimin Li and Hao Wang wrote the paper.

## FUNDING INFORMATION

This study was supported by the Scientific Research Project of Anhui Provincial Education Department (2022AH020079) and the Natural Science Foundation of Anhui Province (2008085MH241).

## CONFLICT OF INTEREST STATEMENT

The authors declare no conflicts of interest.

## Supporting information


**Data S1.** Supporting Information.

**Table S1.** The sequences of siRNAs or shRNAs.

**Table S2.** Primers used in the study.

## Data Availability

The authors declare that all the data supporting the findings in this study are available in this study and its Supplementary materials, or are available from the corresponding author through reasonable request.
