# Supplementary material for: METTL16‐mediated N6‐methyladenosine modification of Soga1 enables proper chromosome segregation and chromosomal stability in colorectal cancer
Source: Cell Prolif. 2023 Dec 12;57(5):e13590. doi: 10.1111/cpr.13590 (PMC11056707; doi:10.1111/cpr.13590)
Supplement: Supplementary file 1 — Data S1. Supporting Information. [file CPR-57-e13590-s001.pdf]

Figure S1

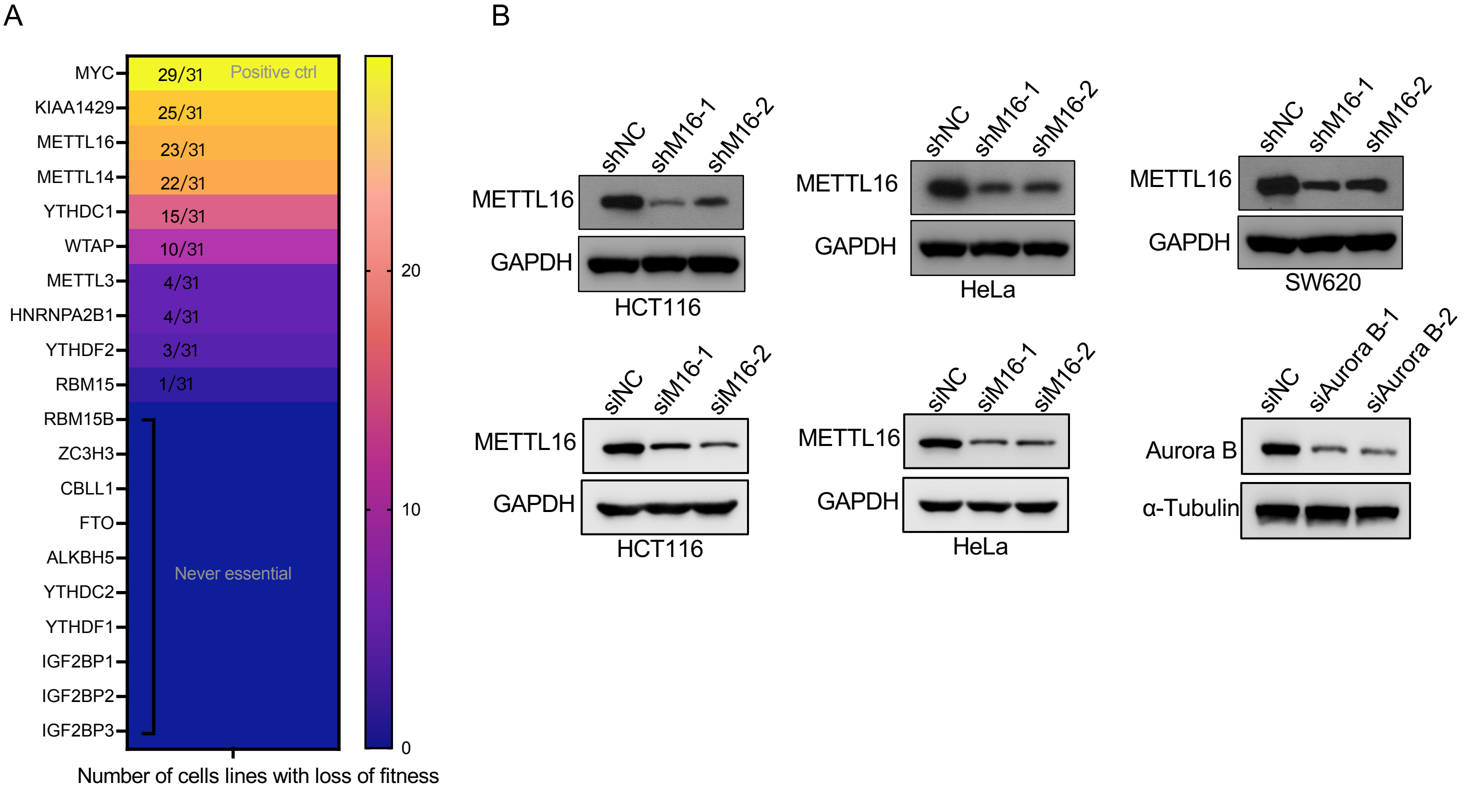

Figure S1. (A) Another set of CRISPR-associated protein 9 knockout screening datasets were analyzed across 31 human colorectal cancer cell lines. The raw data was obtained from https://score.depmap.sanger.ac.uk/. MYC was identified as the positive control, being acknowledged as a promising therapeutic target for cancer. For each gene, the number of essential functions and total CRC cell lines were displayed. For instance, METTL16 (23/31) indicates that knockout of METTL16 displays an essential function in 23 out of 31 cell lines. **(B)** The knockdown efficiency of METTL16 and Aurora B were detected western blotting.

Figure S2

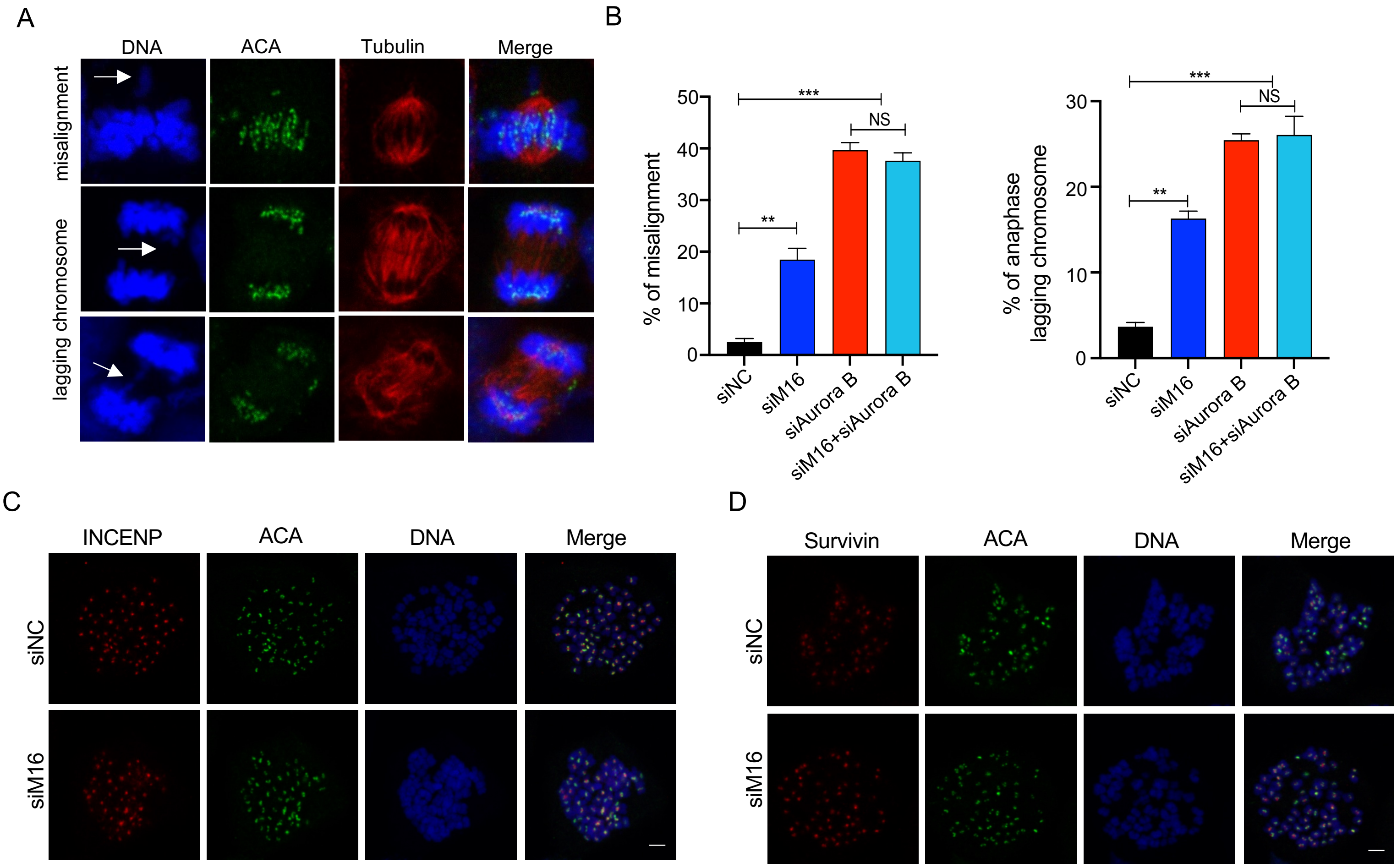

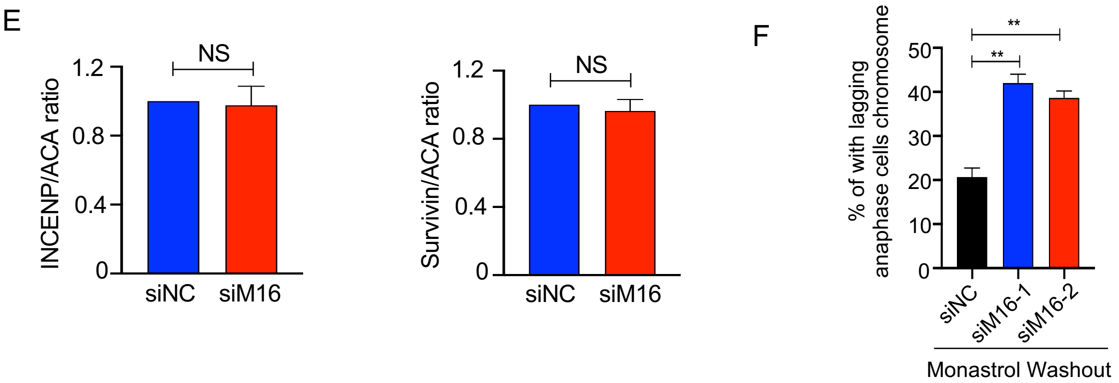

Figure S2. (A-B) Representative immunofluorescence images of siM16 or/and siAurora B in HCT116 cells stained with 4',6-diamidino-2-phenylindole (DAPI), tubulin, and ACA. Quantification of misalignment and Lagging phenotypes in cells transfected with indicated siRNAs. **(C-E)** Chromosome spreads of mitotic cells were stained for INCENP, Survivin, and ACA. The relative signal of INCENP and Survivin was normalized to ACA. Mitotic cells were fixed with 4% paraformaldehyde and stained with indicated antibodies. **(F)** The indicated mitotic cells were incubated with monastrol (50 μM) for 1.5 hr to generate erroneous attachments, and cells were then released into fresh media for another 1 hr before fixation. Errors in chromosome segregation were quantified from anaphase cells. NS, no significance, **, *P*<0.01,***, *P*<0.001 two-tailed t-test. Scale bar, 5 μm.

Figure S3

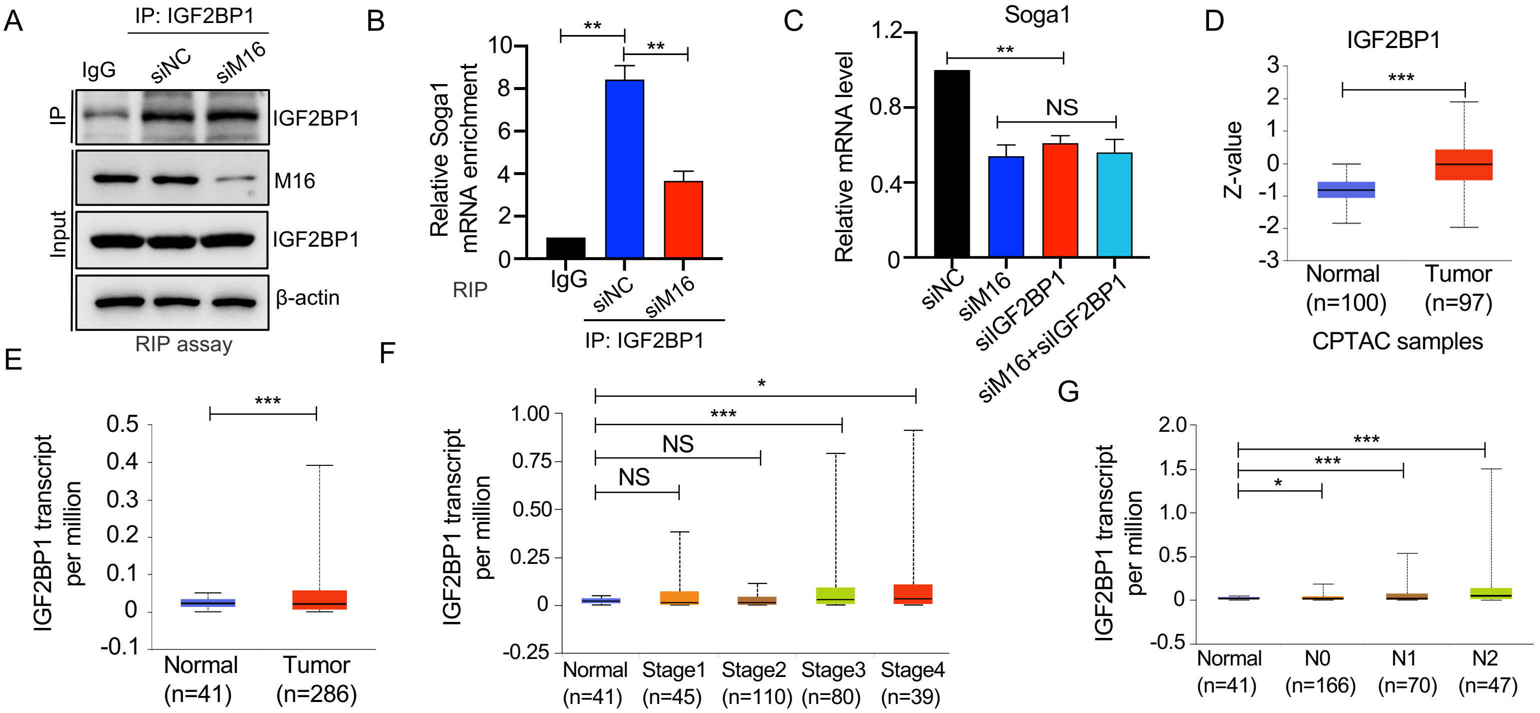

Figure S3. (A-B) RIP-qPCR displayed the relative enrichment of SOGA1 mRNA in each group precipitated with IgG or IGF2BP1 antibody with the normalization to input. IP efficiency of IGF2BP1 was validated using western blotting. **(C)** The mRNA expression of Soga1 was assessed by qRT-PCR following treatment with METTL16 siRNA and/or IGF2BP1 siRNA. **(D)** IGF2BP1 expression in the CPTAC database. **(E-G)** The mRNA level of IGF2BP1 in normal tissues and CRC tissues (E), the association of IGF2BP1mRNA expression with tumor stages (F) and lymph node metastasis (G) in CRC patients in TCGA database (http://ualcan.path.uab.edu/). **, *P*<0.01,***, *P*<0.001 two-tailed t-test.

Figure S4

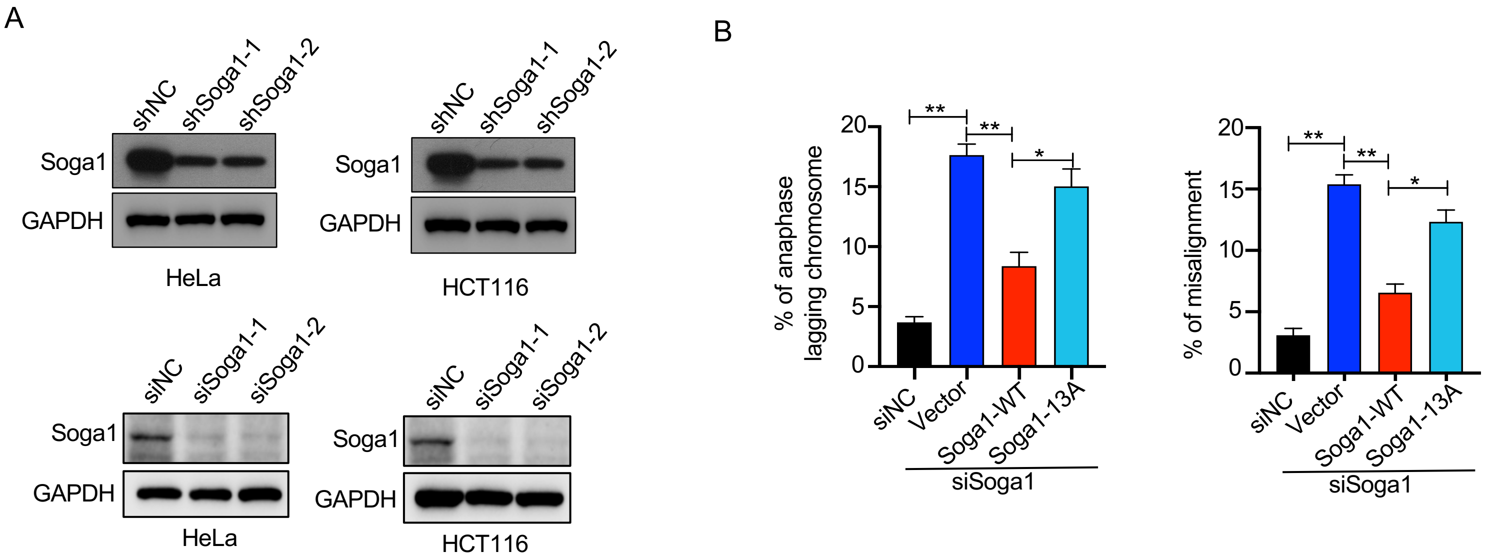

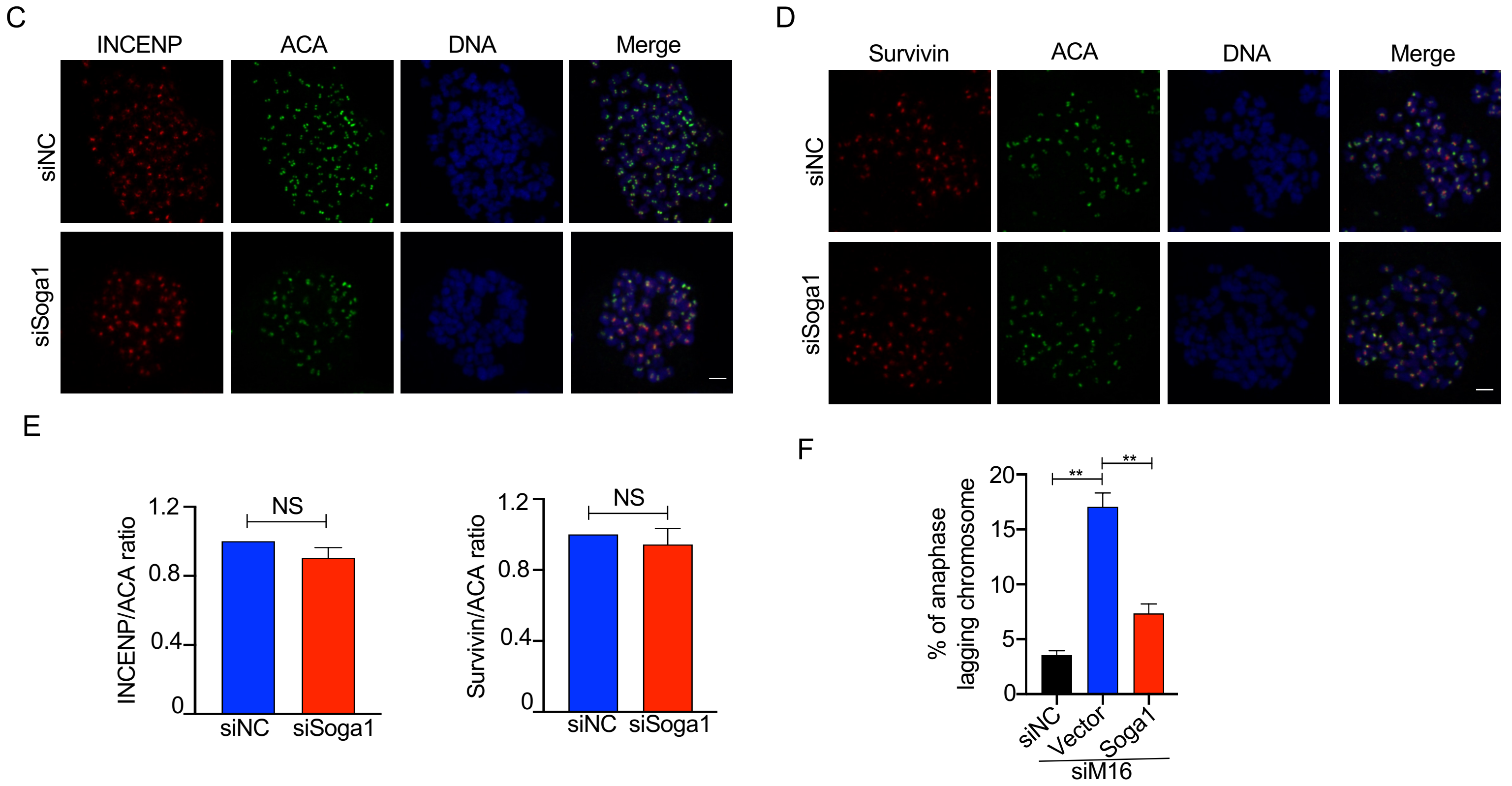

Figure S4. (A) The knockdown efficiency of Soga1 were detected western blotting. **(B)** Percentage of the indicated mitotic cells that display lagging chromosomes or misalignment. An empty vector or a plasmid expressing Soga1-WT or Soga1-13A was introduced into siSoga1 cells. **(C-E)** Chromosome spreads of mitotic cells were stained for INCENP, Survivin, and ACA. The relative signal of INCENP and Survivin was normalized to ACA. Mitotic cells were fixed with 4% paraformaldehyde and stained with indicated antibodies. **(F)** Quantification of lagging chromosome phenotypes in cells transfected with indicated siRNAs or plasmid. NS, no significance,*, *P*<0.05, **, *P*<0.01,***, *P*<0.001 two-tailed t-test. Scale bar, 5 μm

Figure S5

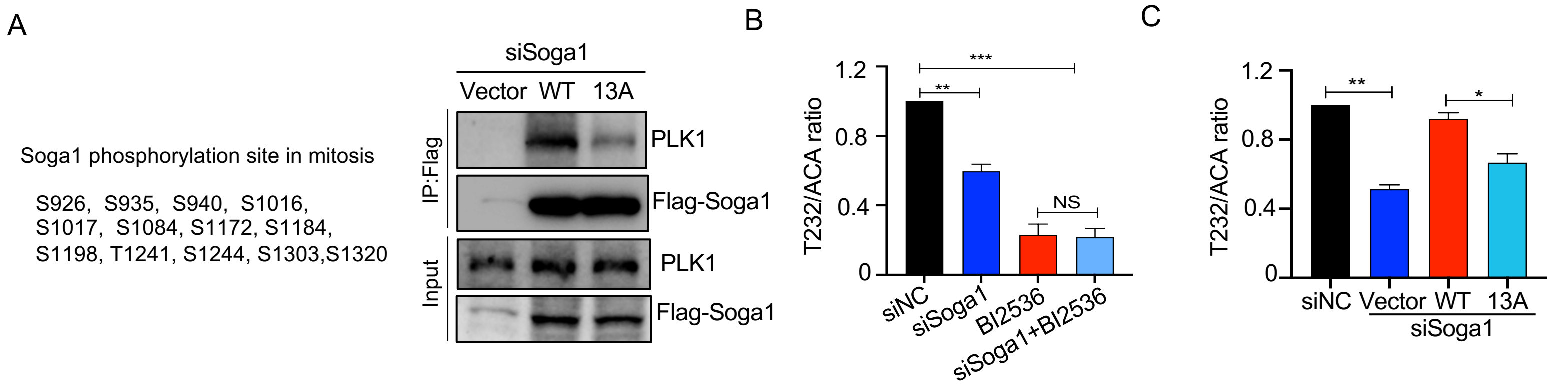

Figure S5. (A) Immunoprecipitation analysis of indicated proteins in cells transfected with the Soga1 siRNA, or the plasmid expressing Flag-Soga1(WT or 13A). Mitotic cells were obtained after nocodazole treatment for 16 h. **(B)** Quantitative analysis of relative intensity of T232/ACA at in cells treated with BI2536 or transfected with indicated siRNAs. **(C)** Quantitative analysis of relative intensity of T232/ACA at in cells transfected with indicated siRNAs or plasmid. NS, no significance,*, *P*<0.05, **, *P*<0.01,***, *P*<0.001 two-tailed t-test.
