# Supplementary material for: METTL16‐mediated N6‐methyladenosine modification of Soga1 enables proper chromosome segregation and chromosomal stability in colorectal cancer
Source: Cell Prolif. 2023 Dec 12;57(5):e13590. doi: 10.1111/cpr.13590 (PMC11056707; doi:10.1111/cpr.13590)
Supplement: Supplementary file 2 — Table S1. The sequences of siRNAs or shRNAs. [file CPR-57-e13590-s002.docx]

| Oligonucleotides | Sequences |
| --- | --- |
| scrambled siRNA | UUCUCCGAACGUGUCACGUTT |
| siIGF2BP2-1 | CAUGCCGCAUGAUUCUUGATT |
| siIGF2BP2-2 | GAACGAACUGCAGAACUUATT |
| siIGF2BP1-1 | CCGGGAGCAGACCAGGCAATT |
| siIGF2BP1-2 | UGAAUGGCCACCAGUUGGATT |
| siIGF2BP3-1 | CGGUGAAUGAACUUCAGAATT |
| siIGF2BP3-2 | GCAAAGGAUUCGGAAACTT |
| siMETTL16-1 | AUGGCUGGUAUUUCCUCGCAATT |
| siMETTL16-2 | GGAAGAUUUUGGACUUUCUTT |
| siSoga1-1 | GUGAGAAGAUCCACGACAA |
| shSoga1-1 | GGAAATGGTTTAAAGAGAA |
| shSoga1-2 | AGAAAGACACCAAGGAGAA |
| shMETTL16-1 | ATGGCTGGTATTTCCTCGCAA |
| shMETTL16-2 | GGAAGATTTTGGACTTTCT |

Table S1. The sequences of siRNAs or shRNAs.
