# Supplementary material for: METTL16‐mediated N6‐methyladenosine modification of Soga1 enables proper chromosome segregation and chromosomal stability in colorectal cancer
Source: Cell Prolif. 2023 Dec 12;57(5):e13590. doi: 10.1111/cpr.13590 (PMC11056707; doi:10.1111/cpr.13590)
Supplement: Supplementary file 3 — Table S2. Primers used in the study. [file CPR-57-e13590-s003.docx]

| Table S2. Primers used in the study. | |
| --- | --- |
| Primers | Sequences（5'-3'） |
| METTL16 forward primer  METTL16 reverse primer  IGF2BP1 forward primer | AGGGAGTAAACTCACGAAATCCT  AACCCCTTGTATGCGAAGCTC  TAGTACCAAGAGACCAGACCC |
| IGF2BP1 reverse primer | GATTTCTGCCCGTTGTTGTC |
| Soga1 forward primer | GAGCAGGATGTCAAGGTCTCT |
| Soga1reverse primer | TTAGCCAGCTCAGTCTCGATG |
| GAPDH forward primer | GGAGCGAGATCCCTCCAAAAT |
| GAPDH reverse primer | GGCTGTTGTCATACTTCTCATGG |
|  |  |
| Soga1 probe | AGCAGGAAGTTGTGCTTGAATTGCT |
| control-probe | AGCAATTCAAGCACAACTTCCTGCT |
